# Asymmetry of Early Endosome Distribution in *C. elegans* Embryos

**DOI:** 10.1371/journal.pone.0000493

**Published:** 2007-06-06

**Authors:** Robert Andrews, Julie Ahringer

**Affiliations:** The Gurdon Institute and Department of Genetics, University of Cambridge, Cambridge, United Kingdom; University of Geveva, Switzerland

## Abstract

**Background:**

Endocytosis is involved in the regulation of many cellular events, including signalling, cell migration, and cell polarity. To begin to investigate roles for endocytosis in early *C. elegans* development, we examined the distribution and dynamics of early endosomes (EEs) in embryos.

**Methodology/Principal Findings:**

EEs are primarily found at the cell periphery with an initially uniform distribution after fertilization. Strikingly, we find that during the first cell cycle, EEA-1 positive EEs become enriched at the anterior cortex. In contrast, the Golgi compartment shows no asymmetry in distribution. Asymmetric enrichment of EEs depends on acto-myosin contractility and embryonic PAR polarity. In addition to their localization at the cortex, EEs are also found around the centrosome. These EEs move rapidly (1.3um/s) from the cortex directly to the centrosome, a speed comparable to that of the minus end directed motor dynein.

**Conclusions/Significance:**

We speculate that the asymmetry of early endosomes might play a role in cell asymmetries or fate decisions.

## Introduction

Early endosomes (EEs) play a role in diverse cellular processes (reviewed in [1]). After endocytosis from the plasma membrane, material is delivered to EEs, the first compartment of the endosomal system. EEs are of particular interest as they are situated at a vesicular ‘cross-roads,’ where endocytosed material can be transported to different compartments, including the late endosome, the recycling endosome, the Trans Golgi Network, or back to the plasma membrane. The route taken depends on the particular cargo being carried, which can be nutrient receptors, activated growth factor receptors, or other types of signalling molecules. To begin to explore functions for endocytosis in *C. elegans* development, we characterised the localization and dynamics of EEs in early embryos.

The *C. elegans* oocyte has no developmentally significant polarity [2]. After fertilization, two meiotic divisions are completed with the extrusion of two polar bodies. Following this, the first mitotic division of the embryo occurs, which is asymmetric, resulting in a larger anterior AB cell and a smaller posterior P1 cell. Asymmetry is established during the first cell cycle, after meiosis, by an unknown centrosome dependent signal to the cortex [2]–[5]. This signal leads to an asymmetry in the actomyosin cytoskeleton which is essential for the asymmetric localization of key polarity regulators, the PAR proteins (reviewed in [6]). In *par* mutants, embryonic polarity is lost and the first cell division is symmetric [7]. In this report, we focus on the distribution of EEA-1 positive EEs during this first embryonic cell cycle, where polarity is established.

## Results and Discussion

### Early endosomes are asymmetrically localised in the early *C.elegans* embryo

**Figure 1:**
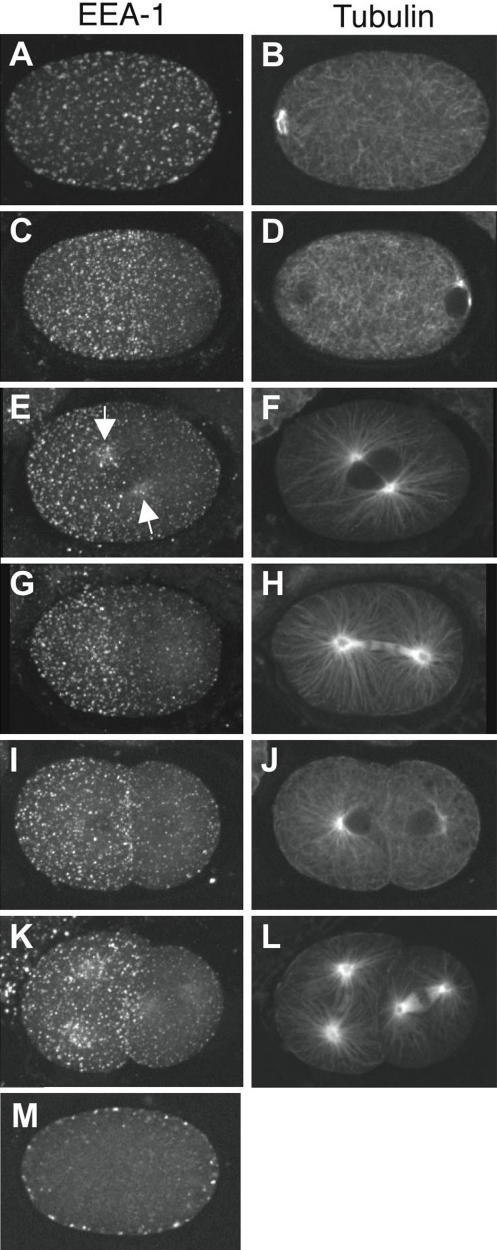
Early endosomes become enriched in the anterior during the first cell cycle. Shown are projections of EEs during the first two cell cycles visualised using anti-EEA-1 and a single focal plane of microtubules visualised using anti-tubulin. (A, B) meiosis. (C, D) polarity establishment; beginning of sperm aster growth. (E, F) prophase; arrows point to enrichment around the asters. (G, H) anaphase. (I, J) Early two cell stage. (K, L) Late two cell stage. (M) central focal plane of embryo in (A,B), showing that EEs are cortically associated. EEs are evenly distributed in meiosis (A), then become depleted from the posterior near the site of the sperm pronucleus (C). Anterior enrichment of EEs is maintained throughout the rest of the cell cycle and in the two cell stage. During meiotic divisions, there is a small clearing of EEs around the meiotic spindle (data not shown). Anterior, left.

We visualised early endosomes (EEs) using the marker EEA1, a Rab5 effector protein that has a role in early endosome docking/fusion [8], [9]. We used a characterized antibody against *C. elegans* EEA-1 that was previously shown to mark early endosomes in other tissues [10]–[13]. In embryos undergoing meiotic divisions (prior to polarity establishment), this revealed a cortically associated layer of puncta symmetrically distributed across the entire embryo surface (Figure 1A, B, M). Individual meiotic EEA-1 positive puncta appear variable in size and shape; the majority are approximately spherical, but there is a significant population of puncta which appear irregularly shaped. It is unclear whether these larger bodies are singular, enlarged EEs, or closely associated aggregates of smaller spherical early endosomes.

Initiation of embryonic polarity is concomitant with growth of sperm asters [3], [14]. As sperm asters become visible, we found that EEA-1 positive EEs begin to be concentrated in the anterior and depleted from the posterior of the embryo (Figure 1C,D). EEs at this stage are also more uniform in size and distribution. The anterior concentration of the EEs becomes progressively more pronounced such that they are highly enriched in the anterior half of the embryo following pronuclear migration (Figure 1E,F). Early endosomes also accumulate around the growing asters, particularly on the anterior-most aster (Figure 1E, arrows). After the first division, the entire cortex of the anterior AB cell and the anterior-most 20–30% of the P1 cortex are enriched for EEs (Figure 1I, J).

**Figure 2:**
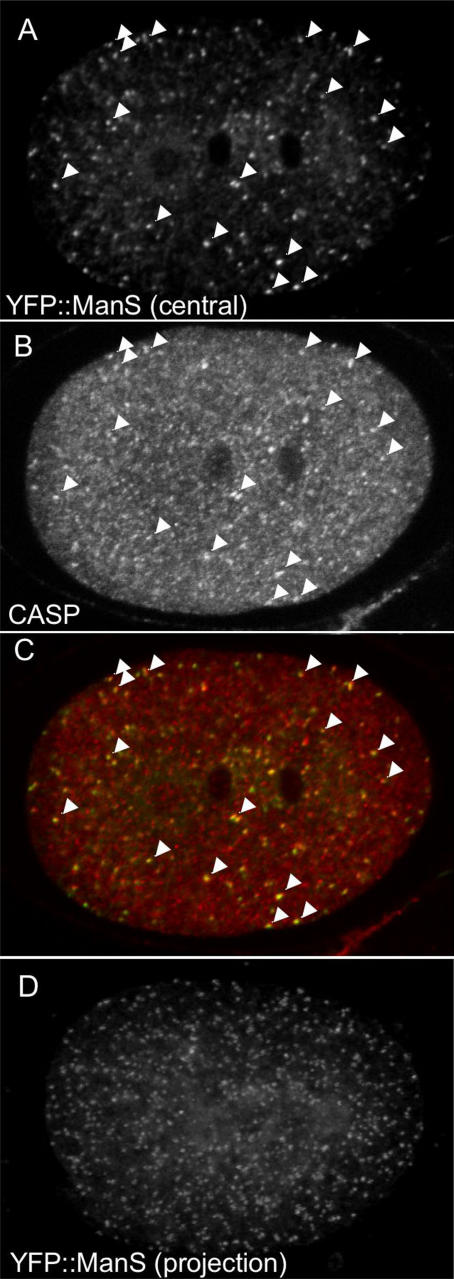
The Golgi shows no asymmetry in distribution during the first cell cycle. (A–C) Even distribution of Golgi structures in a wild-type telophase embryo. The Golgi is visualized using two markers: (A) YFP::ManS, a fusion of YFP and the Golgi resident mannosidase F58H1.1 and (B) anti-CASP/Y54F10AM.4b staining. CASP is an integral Golgi membrane protein [17]. Arrowheads in (A), (B), and (C) (the merged image) show colocalization of puncta with the two markers. Central focal planes are shown. (D) even distribution of Golgi structures in a projection of 12 focal planes of YFP::ManS in a wild-type anaphase stage embryo.

Previous work showed that the endoplasmic reticulum shows a similar anterior enrichment to that seen above for EEA-1 positive EEs [15]. To ask whether anterior enrichment is a general property of endomembrane systems in the early embryo, we examined the distribution of the Golgi compartment. We used a fusion of YFP to a portion of the Golgi resident mannosidase F58H1.1 previously used to mark the Golgi in neurons [16]. Similar to neurons, YFP::ManS primarily labels cytoplasmic puncta in embryos (Figure 2A, D). These puncta show significant co-localization with the integral Golgi membrane protein CASP/Y54F10AM.4b [17], confirming that they are Golgi structures (Figure 2A–C). Unlike the EEA-1 positive EEs, the Golgi puncta show no asymmetry in distribution during the first cell cycle (Figure 2A, D; n = 13). Therefore some, but not all vesicular compartments are asymmetrically localized in the one-celled embryo.

### Early endosome asymmetry requires functional NMY-2 and PAR proteins

The cortically associated anterior polarity proteins PAR-3, PAR-6, and PKC-3 become anteriorly enriched at the same time as the EEA-1 positive EEs [18]–[20]. Other cortically associated proteins such as HMP-1 and POD-1 also show anterior enrichment at this time [21]. The movement of these cortical proteins to the anterior is driven by the asymmetrical contraction of the embryonic actomyosin cytoskeleton. Just prior to polarity establishment, actin and the non-muscle myosin NMY-2 forms a dynamic cortical network of interconnected foci that covers the entire embryo [21]–[23]. In response to the polarity cue from the sperm MTOC, the NMY-2 network locally clears at the posterior and moves towards the embryo anterior [21]. This movement drives the asymmetric localization of a number of cortical proteins, including the PAR-3 complex, to the anterior.

**Figure 3:**
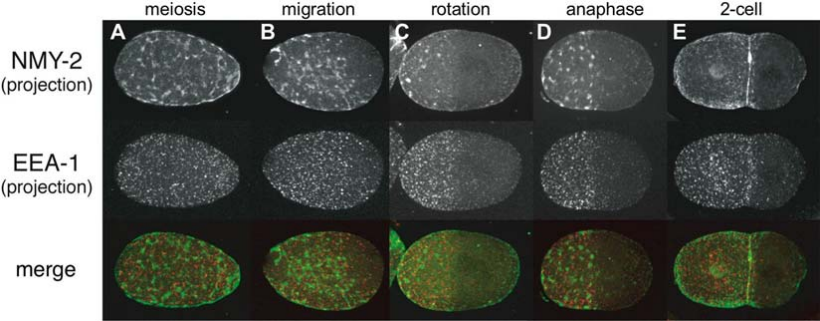
Asymmetry of early endosomes is coincident with NMY-2 asymmetry GFP::NMY-2 embryos co-stained for EEs (anti-EEA-1) and NMY-2 (anti-GFP). The area of enrichment of EEs coinncides with the NMY-2 domain at all stages.

To look at the relationship between EEA-1 positive EEs and NMY-2 asymmetry, we co-stained embryos for EEA-1 and NMY-2. We found that the ‘EE-rich’ domain was coincident with the NMY-2 domain at all stages (Figure 3). The posterior clearings of NMY-2 and EEA-1 are of the same size and location. This suggests that the EE and actomyosin asymmetries may be connected.

**Figure 4:**
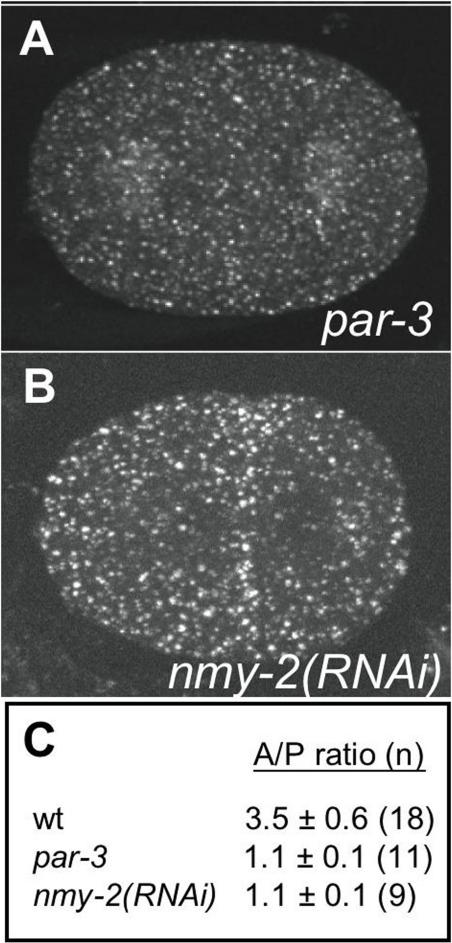
NMY-2 and PAR-3 are required for EE asymmetry. Projections of EEA-1 distributions in (A) *par-3(it71)* mutant, and (B) *nmy-2(RNAi)* embryo. Compare to similar staged wild-type images in Figure 1 (1G and 1I, respectively). Asymmetry is lost in *par-3* mutant and *nmy-2(RNAi)* embryos. (C) Ratio of the number of EEA-1 positive puncta in an anterior region compared to a posterior region of the same size.

To test this idea, we examined EE localisation in embryos where *nmy-2* had been knocked down using RNAi. We found that asymmetry of EEA-1 positive EEs is lost in *nmy-2(RNAi)* embryos; there is an even distribution of EEs across the embryonic cortex throughout the first cell cycle (Figure 4B, C). We also investigated whether establishment of embryonic polarity is required for EE asymmetry by examining their distribution in *par-3* mutant embryos. Similar to *nmy-2(RNAi)* embryos, EE asymmetry is abolished in *par-3* mutant embryos (Figure 4A, C). In both conditions, EEs still cluster around the centrosomes, although the asymmetry of clustering is lost (Figure 4A and data not shown). These results show that EE asymmetry depends on the establishment of embryonic polarity. The requirement for NMY-2 and the cortical colocalisation of NMY-2 and EEs suggest that EE asymmetry may be driven by anterior movement of the actomyosin cortex.

### Early endosomes movement in the early embryo

**Figure 5:**
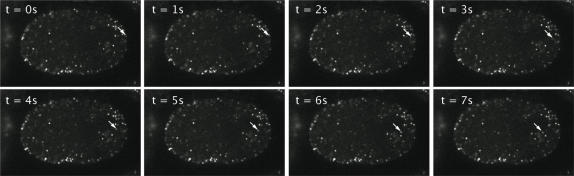
Centrosome directed movement of EEs. EEs visualied live using GFP:EEA-1(FYVE*2). Shown are eight still images taken 1 second apart; arrow points to an EE that moves rapidly towards the centrosome.

It was previously shown that the tandem FYVE domains of EEA-1/T10G3.5 would target GFP to early endosomes in *C. elegans* somatic tissues [24]. To generate a live EE marker for early embryos, we fused this region to GFP and placed it under the control of the germline *pie-1* promoter to generate a GFP::EEA-1(FYVE*2) strain. We made time-lapse movies during nucleocentrosomal rotation of the first division to observe their local behavior and their accumulation around the centrosomes. Most individual cortical puncta moved slowly in what appeared an essentially random manner (mean speed 0.2±0.01 µm/s, n = 10; Movie S1). Of 10 GFP::EEA-1(FYVE*2) puncta followed for 10 seconds, all changed direction of movement at least five times, and movement did not appear to be biased in any particular direction (data not shown). Occasionally, slow-moving cortical EE puncta became highly dynamic and moved rapidly toward the centrosome (mean speed 1.3±0.1 µm/s, n = 11; Figure 5, Movie S1). Once an individual rapidly moving EE arrived at the centrosome it ceased to move and was retained in the growing cluster surrounding the centrosome. The EEs moved in a straight path to the centrosome, suggesting that the movement may be along microtubules. The minus end directed motor dynein would be a candidate for generating such movements since the speed of EE movement measured here is comparable to that of dynein (0.7–1.2 µm/s; [25], [26]).

### Summary

Early endosomes in the *C.elegans* embryo comprise a dynamic compartment. During the first cell cycle, cortically associated EEs become anteriorly enriched. The cortical region enriched with EEs is coincident with the anterior concentration of the non-muscle myosin NMY-2, and both NMY-2 and embryonic PAR polarity is required for EE asymmetry.

Similar to the localization of EEA-1 positive EEs reported here, Poteryaev et al, 2005 showed that the endoplasmic reticulum also becomes anteriorly enriched during the first cell cycle [15]. Furthermore, anterior enrichment of endoplasmic reticulum distribution requires NMY-2, PAR-3, and PAR-6 [15]. In contrast, we found that the Golgi shows no asymmetry in distribution in one-celled embryos indicating that asymmetry is not due to non-specific anterior movement of all membrane systems. The requirement for NMY-2 suggests that the actomyosin cytoskeleton may move the cortical EEs and the endoplasmic reticulum to the anterior. One possibility is that the EEs and the endoplasmic reticulum are linked or associated with the actin cytoskeleton. Anterior movement of the actomyosin cytoskeleton during polarity establishment would result in their anterior enrichment. Consistent with this idea, knockdown of non-muscle myosin *nmy-2* causes the endoplasmic reticulum to have reduced cortical association [15].

We do not yet know the significance of the asymmetry of EEs in early *C. elegans* embryos reported here. It might play a part in promoting the different cell fates of embryonic blastomeres. In *Drosophila*, asymmetry of recycling endosomes has been demonstrated to contribute to cell-fate decisions [27]. Alternatively, this early asymmetry might be a mechanism to provide the AB cell with an appropriate EE load, as it undergoes more cell divisions than P1. Future work interfering with EE function and asymmetry will provide interesting grounds for further study.

## Methods

### Strains

The following strains were used, culturing by standard methods[28]: wild-type Bristol N2; JJ1473 *zuIs45* [*nmy-2*::NMY-2::GFP; *unc-119(+)*] [21]; JA1402 *unc-119(e2498)* III; *weEx55* [Ppie-1:EEA-1(FYVE*2)::GFP; *unc-119*(+)]; JA1410 *unc-119(e2498); weIs18* [*unc-119(+); pie-1p*::ManS::YFP]

### RNA interference

Synthesis of dsRNA and RNAi of *nmy-2* was carried out by injection as in [29] using RNAi feeding clone F20G4.3 (from [30]) as a template for RNAi synthesis. Young adult hermaphrodites were injected, left to recover for 20–21 hours and then embryos dissected for immunofluorescence.

### Immunofluorescence

3-well slides (75×25 mm, Cell-line Associates, catalogue No10-2066) were coated with 0.3% poly-Lysine (Sigma, P-1524). Gravid hermaphrodite worms were washed and then transferred to a 7 µl drop of egg buffer in a poly-lysine coated well. Embryos were released from the worms by cutting them with a 23-gauge needle and then covered with a 22×40 mm coverslip, which compresses the embryos. The whole slide was frozen on dry ice for 10 minutes. The coverslip was then quickly removed and the slide fixed in methanol at room temperature for 20 minutes. Samples were washed and re-hydrated with a 5 min wash in PBS followed by two 10 minute washes in PBS+0.2% Tween-20 (PBS-T), and then incubated with primary antibody at 4°C overnight. Slides were washed three times for 10 minutes in PBS-T, incubated for 1 hour at 37°C with secondary antibody and DAPI, washed a further three times in PBS-T and then mounted in Mowiol. Antibodies and dilutions are shown below. Rabbit anti-EEA-1[10], [11] used 1:50 was kindly provided by Barth Grant. The antibody is specific for EEA-1 as RNAi of *eea-1* results in loss of all staining (not shown). Anti-CASP/Y54F10AM.4b was a gift from A. Gillingham and S. Munro. Mouse anti-tubulin from Sigma (clone DM1 A1) was used at 1:100, and chicken anti-GFP from Chemicon was used at 1:200. Secondary antibodies were from Jackson Immunoresearch.

### Construction of *pie-1*::EEA-1(FYVE*2)::GFP and *pie-1*::YFP::ManS

The 2xT10G3.5(FYVE) sequence described in [24] was PCR amplified from a clone kindly provided by F. Muller and Gateway cloned into pDONR201 (Invitrogen). The 2xT10G3.5(FYVE) sequence was transferred, again by Gateway cloning, to pID3.01B, which contains the germline *pie-1* promoter upstream of GFP, a gateway cassette, and *pie-1* 3′ sequences, as well as the *unc-119*(+) gene for use as a selection marker (kindly provided by G. Seydoux). *pie-1*::YFP::ManS was constructed by PCR amplifying and Gateway cloning the YFP fusion to 82aa of ManS [16] into pDONR201, then transferring this to pID2.02, which contains germline *pie-1* promoter upstream of a gateway cassette and *pie-1* 3′ sequences, as well as the *unc-119*(+) gene for use as a selection marker (kindly provided by G. Seydoux). Transgenic lines containing these reporter constructs were made by microparticle bombardment [31].

For live analyses of EE movement, embryos carrying *pie-1*:GFP::EEA-1(FYVE*2) were mounted in egg buffer (118 mM NaCl, 40 mM KCl, 3 mM CaCl2, 3 mM MgCl2, 5 mM HEPES pH 7.2) on 18×18 mm coverslips coated with 0.3? poly-Lysine (Sigma, P-1524) and the coverslips inverted onto 3% agar pads and sealed with petroleum jelly. Recordings were done using a Perkin-Elmer Spinning disk confocal microscope. Images were captured at one second intervals. Positions of individual EE puncta were tracked using Improvision Openlab software over 10–20 frames for slow moving puncta and for 5–10 frames for rapidly moving puncta. For each individual EE tracked, speed was calculated between one second frames and then speeds averaged.

## Supporting Information

Movie S1 Movement of early endosomes in a one-celled embryo. Rapid centrosome directed movement of early endosomes in a one-celled embryo. Pairs of DIC and GFP images were taken every 1 sec (140 sec total recording). In this central focal plane several puncta can be seen moving towards the centrosomes, which are associated with the pronuclei (visible in the DIC image). The anterior cortex shows more EEs than the posterior cortex although anterior enrichment is not yet complete; the time scale of the movie is too short to see anterior movement of cortical EEs.
(5.10 MB MOV)
